# Using Classroom Data to Teach Students about Data Cleaning and Testing Assumptions

**DOI:** 10.3389/fpsyg.2012.00354

**Published:** 2012-09-25

**Authors:** Kevin Cummiskey, Shonda Kuiper, Rodney Sturdivant

**Affiliations:** ^1^Department of Mathematical Sciences, United States Military AcademyWest Point, NY, USA; ^2^Department of Mathematics and Statistics, Grinnell CollegeGrinnell, IA, USA

**Keywords:** Guided Interdisciplinary Statistics Games and Labs, messy data, model assumptions

## Abstract

This paper discusses the influence that decisions about data cleaning and violations of statistical assumptions can have on drawing valid conclusions to research studies. The datasets provided in this paper were collected as part of a National Science Foundation grant to design online games and associated labs for use in undergraduate and graduate statistics courses that can effectively illustrate issues not always addressed in traditional instruction. Students play the role of a researcher by selecting from a wide variety of independent variables to explain why some students complete games faster than others. Typical project data sets are “messy,” with many outliers (usually from some students taking much longer than others) and distributions that do not appear normal. Classroom testing of the games over several semesters has produced evidence of their efficacy in statistics education. The projects tend to be engaging for students and they make the impact of data cleaning and violations of model assumptions more relevant. We discuss the use of one of the games and associated guided lab in introducing students to issues prevalent in real data and the challenges involved in data cleaning and dangers when model assumptions are violated.

## Introduction

The decisions that researchers make when analyzing their data can have significant impacts on the conclusions of a scientific study. In most cases, methods exist for checking model assumptions, but there are few absolute rules for determining when assumptions are violated and what to do in those cases. For example, when using *t*-tests or ANOVA, decisions about normality, equal variances, or how to handle outliers are often left to the discretion of the researcher. While many statistics courses discuss model assumptions and data cleaning (such as removing outliers or erroneous data), students rarely face data analysis challenges where they must make and defend decisions. As a result, the impacts of these decisions are rarely discussed in detail.

The topics of data cleaning and testing of assumptions are particularly relevant in light of the fact that there have been several high-profile retractions of articles published in peer-reviewed psychology journals because of data related issues. In June 2012, Dr. Dirk Smeesters resigned from his position at Erasmus University and had a paper retracted from the *Journal of Experimental Psychology* after it was determined his data was statistically highly unlikely. He admitted to removing some data points that did not support his hypothesis, claiming that this practice is common in psychology and marketing research (Gever, 2012). Simmons et al. (2011) show that even when researchers have good intentions, they control so many conditions of the experiment that they are almost certain to show statistically significant evidence for their hypothesis in at least one set of conditions. These conditions include the size of the sample, which outliers are removed, and how the data is transformed. They argue that the ambiguity of how to make these decisions and the researcher’s desire to obtain significant results are the primary reasons for the large number of false positives in scientific literature (Simmons et al., 2011).

Replication studies are designed to ensure the integrity of scientific results and may help detect issues associated with the data and model assumptions. However, replication is not a panacea. Miller (2012) shows that the probability of replicating a significant effect is essentially unknowable to the researcher so the scientific community may not always correctly interpret replication study results. Furthering the difficulty in assessing the quality of researchers’ decisions involving data is the fact that relatively few replication studies are done on published research. Journals prefer to publish original research and rarely publish replications of previous studies even if the replication shows no effect. Therefore, there is little incentive for researchers to replicate others’ results which increases the likelihood that studies resulting in false positives are accepted into scientific journals. Dr. Brian Nosek and a group working on the ambitious Reproducibility Project are attempting to replicate every study published in three major psychology journals in 2008 (Barlett, 2012).

Through student generated datasets, we demonstrate how students in the same class, with the same raw dataset, and using the same statistical technique can draw different conclusions without realizing the assumptions they made in their analysis. There is much truth to Esar’s (1949) humorous saying “Statistics [is] the only science that enables different experts using the same figures to draw different conclusions.” Instead of having our students believe that statistics is simply a set of step-by-step calculations, we emphasize the influence a researcher’s judgment can have on study conclusions.

Reforms in statistics education encourage an emphasis on understanding of concepts, interpretation, and data analysis instead of formulas, computation, and mathematical theory (Garfield et al., 2007; DeVeaux and Velleman, 2008). Curricula based on these reforms move away from teaching statistics as a collection of facts. Instead, they encourage the scientific process of interdisciplinary data analysis as statistics is actually practiced. Paul Velleman states, “It seems that we have not made [this] clear to others – and especially not to our students – that good statistical analyses include judgments, and we have not taught our students how to make those judgments” (Velleman, 2008). Our classroom activities and corresponding datasets demonstrate the importance of emphasizing these points and offer ideas for those teaching courses involving data analysis and experimental design to introduce the discussion in the classroom.

## The Tangrams Game and Lab

**Figure 1:**
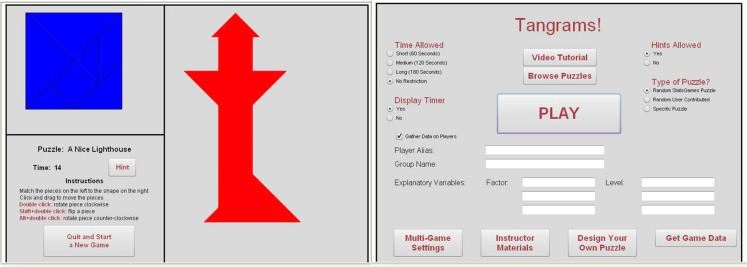
**Tangrams web interface**.

Tangrams is an ancient Chinese puzzle where players arrange geometrically shaped pieces into a particular design by flipping, rotating, and moving them. The online Tangrams game and the web interface, shown in Figure 1, allow students the opportunity to play many versions of the original game.

Prior to starting the game, the class decides upon one or more research questions they want to investigate as a group. For example, students may decide to test whether the game completion time depends on the type of music played in the background, or they could test if one gender is more likely to use hints. Students then design the experiment by determining appropriate game settings and conditions for collecting the data. After the student researchers design the experiment, they become subjects in the study by playing the game. The website collects the players’ information and records their completion times. The data is available for immediate use through the website. If one research study is designed for the entire class, every student plays the game under similar conditions and a large sample of data is immediately available through the website for analysis. The students return to their role of researcher using the data that they just collected.

Next, students (as a class or in small groups) make decisions about data cleaning, check assumptions, perform a statistical test of significance, and state their conclusions. Classroom testing of the Tangrams game and associated labs over the last three semesters has given us a rich data set demonstrating the impacts of data cleaning and the importance of validating the assumptions of statistical tests.

The Tangrams game-based lab gives students exposure to the entire research process: developing research questions, formulating hypotheses, designing experiments, gathering data, performing statistical tests, and arriving at appropriate conclusions. This lab is a fun and effective way for instructors to transition from textbook problems that focus on procedures to deeper learning experiences that emphasize the importance of proper experimental design and understanding assumptions.

## Impacts of Data Cleaning

**Figure 2:**
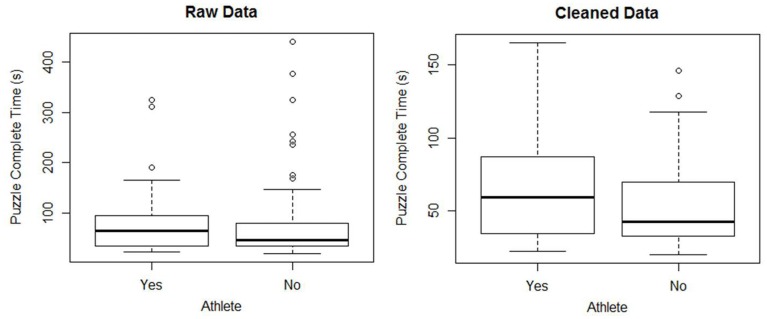
**Side-by-side boxplots of the completion time of the Tangrams game for raw and cleaned data**.

Figure 2 shows boxplots of data collected at West Point in the fall semester of 2011 for one research question. The dependent variable is the time to successfully complete a specified Tangrams puzzle. The independent variable is athlete (Yes means the student plays on a collegiate team while No means the student does not play on a collegiate team). Students were given an overview of the game by their instructor and then allowed to practice the game once on a different puzzle in order to get familiar with the game controls. For both groups, the distributions of completion times appear unimodal with a large positive skew. There are several outliers present within the dataset. Discussing the data with students tends to provide the following reasons for at least some of the very high times:

1. The student did not fully understand the object of the game even after the practice game.
2. The student did not fully understand how to manipulate the pieces even after the practice game (some puzzle shapes require a piece to be flipped while others do not).
3. The full attention of the student was not on the game during the entire time recorded.

Any one of these reasons could justify removing that observation from the analysis. Before conducting their analysis, some students removed the positive outliers shown in the boxplots of the raw data in Figure 2,^1^ (Carling, 2000; Ueda, 2009). For the rest of this paper, we will refer to the data set after removing these outliers as the cleaned data. Through class discussion of the data after the experiment, students recognized issues with the conduct of the experiment and the importance of understanding the data collection mechanism. They were able to formulate recommendations for improving the future experiments such as better control of extraneous variables and including a method for getting feedback from players to determine if their results were erroneous.

The decision on whether or not to keep outliers or erroneous data in the analysis has a very clear impact on the results. For example, Table 1 shows that removing the outliers identified within the boxplots in Figure 2 can change the *p*-value from 0.478 to 0.058 for a one-way ANOVA. Most students found the difference in *p*-values surprising, especially given that the sample sizes of both groups are larger than 30. Many researchers would interpret a *p*-value of 0.058 as being small enough to conclude that there is some evidence that there is a difference between the two population means. This conclusion is clearly different than the one we would reach with all the data points.

**Table 1:** **Summary statistics for raw and cleaned data**.

	Raw data		Cleaned data (outliers removed)	
	Athlete	Non-athlete	Athlete	Non-athlete
Sample size	36	92	33	84
Sample mean	82.72	72.50	65.23	53.02
SD	72.00	73.50	39.35	27.11
	***p*-value = 0.478** (one-way ANOVA on difference in means)		***p*-value = 0.058** (one-way ANOVA on difference in means)	

## Impacts of Invalid Model Assumptions

In addition to considering impacts of cleaning data, results of these classroom experiments show the impact of model assumptions on the conclusions of the study. The two sample *t*-test and one-way ANOVA are both parametric hypothesis tests used to determine if there is a difference between the means of two populations. In our case, we want to see if the difference between the means of the athletes and non-athletes is statistically significant. The null (*H*_0_) and alternate (*H_a_*) hypotheses are:

$$\begin{array}{rcll} H_{0}:\mu_{\text{A}}=\mu_{\text{N}} & & & \\ H_{a}:\mu_{\text{A}}\neq \mu_{\text{N}} & & & \end{array}$$

where μ_A_ and μ_N_ are the means of the athlete and non-athlete populations. Both tests assume that we have random samples from their respective populations and that each population is normally distributed. The one-way ANOVA also assumes equal variances. However, the two-sample *t*-test can be conducted without the equal variance assumption (sometimes called Welch’s *t*-test). In this section, we will discuss the equal variance and normality assumptions.

Some texts suggest that formal tests should be used to test for equal variances. However, some tests, such as Bartlett’s test (and the *F*-test), are very sensitive to non-normality. Even with the outliers removed, the cleaned data is still strongly skewed right (see Figure 2). Box criticized using Bartlett’s test as a preliminary test for equal variances, saying “To make the preliminary test on variances is rather like putting to sea in a rowing boat to find out whether conditions are sufficiently calm for an ocean liner to leave port” (Box, 1953). Levene’s test of homogeneity of variance is less sensitive to departures from normality (Levene, 1960; Brown and Forsythe, 1974). For the cleaned data, Levene’s test gives a *p*-value of 0.023, indicating there is evidence of unequal variances.

A commonly used informal test is to reject the equal variance assumption when the ratio of SD (largest SD over the smallest SD) is greater than 2 (Moore and McCabe, 2003). Using this rule of thumb on our data, the ratios for the raw and cleaned data are 1.02 and 1.45, respectively. Using this informal rule, we fail to reject the assumption of equal variances for both the raw and cleaned data.

Whether or not the researcher decides to assume the two populations have equal variances will contribute to the choice of which statistical test to perform and has a surprisingly large impact on the *p*-value of the two sample *t*-test. Without assuming equal variances, the *p*-value of the two sample *t*-test on the cleaned data is 0.109, which is considerably larger than the *p*-value of 0.058 found when assuming equal variances. Note that the *p*-value for the *t*-test assuming equal variances and the one-way ANOVA are mathematically equivalent and result in the same *p*-value.

To explain the impacts of this equal variance assumption, we need to recognize the influence of unequal sample sizes. When the group with the smallest sample size has a larger SD, the mean square error (or pooled SD) is likely to underestimate the true variance. Then ANOVA is likely to incorrectly reject the null hypothesis (conclude that there are differences when there really are no differences between group means).

A second assumption that a researcher should validate is that both samples are from normally distributed populations. From the boxplots in Figure 2, a student should suspect that the population distributions are not normal. Additional tools such as histograms and normal probability plots clearly show that the sample data is not normally distributed. For both athletes and non-athletes, the Shapiro–Wilks test for normality rejects the null for both samples with *p*-values less than 0.001, providing further evidence that the assumption of normality is not valid (see Table 2 for a summary of the results of various Shapiro–Wilks tests).

**Table 2:** **Summary of Shapiro–Wilks normality test under various conditions**.

	Athlete	Non-athlete
Raw data	<0.001	<0.001
Cleaned data	0.00157	<0.001
Log-transformed raw data	0.118	<0.001
Log-transformed cleaned data	0.153	0.0521

**Figure 3:**
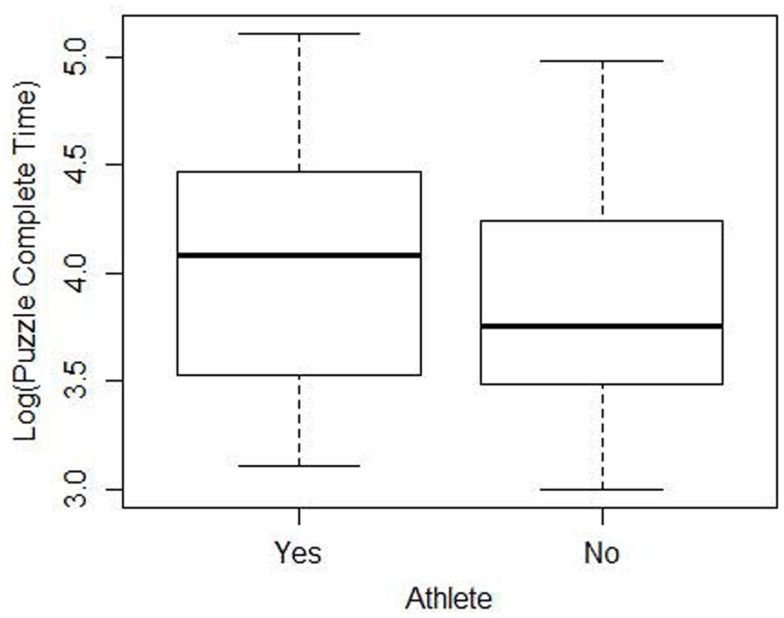
**Side-by-side boxplots of the log of completion time for the cleaned data**.

When faced with data that indicates the normality assumption is not valid, transforming the data is one method to allow the analyst to proceed with the analysis. In this case, taking the log of the completion times results in plots that appear much closer to the shape of the normal distribution^2^. Figure 3 shows a boxplot of the cleaned data after the log transformation. It is more appropriate to conduct the statistical test using the transformed data since the normality assumption is more closely met. When the two sample *t*-test (unequal variances) is performed on the transformed data, the resulting *p*-value is 0.307 which would lead us to conclude that there is not a significant difference between athletes and non-athletes. These results are somewhat different from the *p*-value of 0.478 obtained using the raw data, although the difference in the *p*-value did not change the conclusion of the test.

Researchers using the cleaned data face similar issues. Even after removing the outliers, the cleaned data still is strongly skewed to the right. Once again, a log transform improves the normality assumption. Conducting the two sample *t*-test on the cleaned log transformed data results in a *p*-value of 0.18.

We have shown that even when students start their analysis with the same raw dataset, decisions involving data cleaning and validation of model assumptions cause *p*-values to vary between 0.058 and 0.478. Table 3 summarizes the different *p*-values based on the assumptions we discussed in the last two sections. This clearly demonstrates that model assumptions need to be checked before any statistical conclusions are drawn. It also shows that a researcher determined to find significant results can do so by choosing a set of assumptions resulting in the smallest *p*-value.

**Table 3:** **Summary of *p*-values for the difference in means between Tangrams completion times of athletes and non-athletes under various assumptions**.

	Raw data (*p*-value)	Cleaned data (*p*-value)
Two-sample *t*-test assuming equal variances	0.478	0.058
Two-sample *t*-test without assuming equal variances	0.475	0.109
Two-sample *t*-test assuming equal variance using the log-transformed data	0.307	0.139
Two-sample *t*-test assuming equal variance using the log-transformed data	0.323	0.180

In introductory statistics courses, this dataset can be used to focus on tests that are based on the equal variance assumption and the normality assumption (t-tests and ANOVA) and how the violation of these assumptions can influence *p*-values as shown in Table 3. However, there are several other statistical techniques that are typically beyond the scope of an introductory course that can be discussed with this dataset. In addition to Levene’s test and the Shapiro–Wilks test shown above, instructors could discuss the following:

- More advanced methods, such as the Box-Cox power transformation, can be used to find a better transformation (Osborne, 2002; Olivier and Norberg, 2010).
- Response time is often modeled with the exponentially modified Gaussian distribution (ex-Gaussian distribution). This game typically provides a relevant example of data that is better modeled with a distribution other than the normal distribution (Marmolejo-Ramos and González-Burgos, 2012).
- Many students believe that if the sample size is greater than 30, the Central Limit Theorem guarantees that tests based on the normal distribution are appropriate to use. Tim Hesterberg has written an easy-to-understand article that is freely available online that challenges this assumption (Hesterberg, 2008). Having students read this article helps them understand that while the “sample size greater than 30” is a nice guideline, it is not an absolute rule.
- The data can be used to demonstrate better tools to enhance the visibility of data that is not normally distributed. For example, the shifting boxplot and the violin plot with confidence interval around the mean provide additional information not displayed in the basic boxplot (Marmolejo-Ramos and Matsunaga, 2009; Marmolejo-Ramos and Tian, 2010).
- The analysis of the Tangrams data is equally suited for courses that emphasize other concepts, such as Bayesian approaches to data analysis (Kruschke, 2011).
- The data can be analyzed with techniques that are not based on the normality assumption, such as randomization and permutation tests.

## Random Sampling

In our experience, the assumptions of statistical tests such as those discussed in the previous section are at least covered in typical statistics textbooks and classes. An assumption that is addressed far less often but that should always be validated before any statistical conclusions are drawn about populations is that each observation is a sample randomly selected from the population. Homework-style problems do not give enough information about how the data was collected for students to consider the quality of the sample. When students conduct their own experiment, the students are more aware of this issue in the resulting data.

Some sources of bias are easily identifiable. For example, this sample gathered at West Point is most certainly not generalizable to all colleges nationwide, as only about 15% of cadets are female. In addition, it is important to discuss the impact of a researcher acting as a subject within their own research study. Other sources of bias are not so easily identifiable but warrant discussion. This is especially the case with observational studies such as ours where the students enrolled in the course are acting as subjects in the study. For example, it could be possible that there are other factors that the athletes in our sample share that were the true reason for differences in their times when playing the puzzle game. One possibility is that the athletes have early morning practice and are therefore more tired than the non-athletes. Also, because students decided and knew which variables were being investigated, there is the possibility of stereotype threat, where groups are primed to perform better or worse.

From our experience, the following discussion questions are effective at addressing some of the important issues involved with random sampling and designing experiments.

- If you had a chance to play the game under different circumstances, would you be able to perform better? Describe any factors that may have kept you from doing your best while playing the game.
- Do you think outside factors (like the ones you or others mentioned in the previous question) could impact the results of a study? Should researchers provide some type of motivation for their subjects in various studies to do their best?
- If you were conducting this study again, how would you control for any key factors that may influence each subject’s performance?
- Ask small groups to design their own study, addressing other not so obvious conjectures.
  - Are there any variables (such as academic major or gender) that explain why some players solve Tangrams puzzles faster than others?
  - What type of student improves the most when playing this type of game?
  - Is it helpful for a second student to provide hints?

## Additional Data Sets

The Tangrams game and corresponding labs are designed to be flexible so that students can design studies related to their interests. In another semester at West Point, we investigated the relationship between academic major and Tangrams performance. Students majoring in math, science, and engineering disciplines (MSE) were compared to those majoring in other disciplines (non-MSE). In this study, the raw data resulted in a *p*-value of 0.353 for the two sample *t*-test. When outliers were removed from the data, the *p*-value decreased to 0.071. Other classes have tested two-way or three-way factorial designs. For any variables that students are interested in testing, the nature of the Tangrams game tends to produce positively skewed data and outliers. Thus, any study conducted by students with this game provides opportunities to demonstrate the importance of data cleaning and model assumptions. Table 4 contains a list of suggested independent variables that could be used to explain Tangrams performance.

**Table 4:** **Candidate independent variables to explain Tangrams performance**.

Variable	Research question
Gender	Do males or females perform better at tangrams?
Academic major	Do students majoring in science, technology, engineering, and mathematics perform better at tangrams than other students?
Type of high school attended	Do students who attended private or public high schools perform better at tangrams?
Athlete	Do college athletes perform better at tangrams than non-athletes?
Political affiliation	Do students who affiliate with the democratic, republican, or other parties perform better at tangrams?
Academic performance	Do students who made the dean’s list perform better at tangrams than those that did not?

In this paper, we focused on the time to complete the puzzle as the response or dependent variable. The Tangrams game offers many more dependent variables that can be investigated. Table 5 contains a list of some of the other dependent variables that can be investigated using Tangrams.

**Table 5:** **Candidate dependent variables**.

Variable	Description
Puzzle completion time	Time to complete a tangrams puzzle
Puzzle success or failure	Given a fixed amount of time, whether or not a student can complete the puzzle
Number of moves	Number of moves (a flip or rotation) required to solve the puzzle
Time to quit	Time before a student quits a puzzle that is impossible to solve
Time to receive a hint	Time until a student asks the game for a hint
Number of puzzles solved	Given a fixed amount of time, the number of puzzles that a student can solve

In more advanced courses, small groups of student researchers have used these online games to develop more complex research studies. For example, one group built upon Butler and Baumeister’s (1998) findings that a friendly observer (compared to neutral or hostile observers) had a detrimental effect on the ability of participants to accurately complete difficult tasks. They conducted a study with a repeated measures design to determine whether this effect would be the same if the friendly observer actively offered advice on how to solve Tangrams puzzles.

We have developed a series of online games and associated labs like the one discussed in this paper. Multiple independent variables can be used to test memory or spatial reasoning skills. Students can choose variables (such as using hints, amount of time allowed, or number of pieces) that are very likely to result in small *p*-values even with small sample sizes. Other independent variables, such as gender or major, are less likely to have small *p*-values. When multiple studies are conducted, we typically find no more than one or two of the studies will show significant differences among gender. This can lead to very interesting discussions of publication bias that can occur when only research studies with small *p*-values are published. Each game-based lab or research project allows for quick, anonymous, and automated data collection that can be used to demonstrate the importance of impacts of data cleaning and model assumptions on the results of a study.

## Student Comments and Assessment

In statistics education, it is often challenging to have students experience the true complexities of conducting a large research study. Designing an experiment, collecting and analyzing data, and deciding upon and carrying out the appropriate statistical test are usually too time-consuming, costly, or impractical for an introductory statistics course. As a result, most students learn statistical calculations without a tie to the context of the scientific research and become disinterested in, and even cynical toward, statistics. An alternative to lectures and textbook style problems is to incorporate research-like experiences in the classroom.

The Classroom Undergraduate Research Experience (CURE) survey of undergraduate science evaluated courses that contain research-like experiences. Research-like experiences are activities that contain “Group work, reading primary literature, data collection and analysis… students conduct research in which the outcome is not known (even to the course instructor) and students have at least some input into the research topic and design of the methodological approach.” Results of the CURE survey show that “Students in high research-like courses report learning gains similar in kind and degree to gains reported by students in dedicated summer research programs” (Lopatto, 2010). In addition, Wei and Woodin (2011) found “Evidence is emerging that these approaches are introducing more underrepresented minorities to scientific research in the classroom.” These game-based labs are designed so that, with technology, students can gain many of the benefits of research-like experiences.

A formal assessment of student attitudes and learning was conducted throughout the 2011–2012 school year. These materials were taught in five sections of an introductory statistics course. When we asked students what they liked best about the lab, typical responses were:

- The data set was real and we played a part in creating it.
- To be able to see an actual scenario where what we learned can be used.
- The fact that we could collaborate…
- …work at own pace, ask questions as needed.
- I liked that getting the data was very quick and easy.
- Playing the game!

As a group, students enjoyed playing the games. Even though the online game is fairly simple with plain graphics, it was considered a welcome break from normal classroom activities. The level of interest in trying to explain potential biases in Tangrams performance was very high. Ideas ranged from number of hours of sleep to SAT scores to the age of the player. This activity also seemed to truly engage students who were otherwise quiet throughout the semester.

Many students commented that they liked using “real” data for the *first* time in their course. This comment came as a surprise because the instructors had used data from economics, sports, scientific, and military websites in lessons prior to this lab. However, to the students, if they are not involved in the collection of the data, it is not real to them. Involving students in data collection makes them much more interested in the outcome of a statistical process. In addition, messy data makes the decision process more real to students.

In many courses using the lab, students had yet to actively experience the context for the statistical procedures they were learning. They had only seen textbook type questions that give them the research question, the experiment, the data, and the statistical procedure to use. After completing the lab, many students commented that they saw how statistical procedures are actually used by people outside the statistics classroom. Survey results suggest that students enjoyed the lab and felt like they had learned from the experience. In this assessment, 81% of students either agreed or strongly agreed that the Tangrams lab was a good way of learning about hypothesis testing, while only 10% disagreed. seventy-four percent either agreed or strongly agreed that the Tangrams lab improved their understanding of using statistics in research. Complete results of the survey are displayed in Table 6.

**Table 6:** **Survey results for 115 students after completing the Tangrams lab**.

Survey question	Strongly agree (%)	Agree (%)	Neutral (%)	Disagree (%)	Strongly disagree (%)
The Tangrams lab was a good way of learning about hypothesis testing	43	38	8	7	3
Students who do not major in science should not have to take statistics courses	5	10	23	37	24
Statistics is essentially an accumulation of facts, rules, and formulas	10	34	30	19	6
Creativity plays a role in research	30	47	12	7	4
If an experiment shows that something does not work, the experiment was a failure	9	2	5	31	52
The tangrams lab had a possible effect on my interest in statistics	17	38	32	13	1

Although our students have laptop computers and are required to bring them to class, the Tangrams lab has been implemented in other classroom conditions. In large sections, where each student does not have a laptop, students can play the game outside of class in preparation for the next class period. If no computers are available in class, the guided labs that we have available are detailed enough to allow students to do most of the computational work outside the classroom, where most students presumably have access to a computer. The instructor can then use class time to discuss results and interpretations of findings.

## Conclusion

Online games and guided labs such as Tangrams are fun and effective ways to incorporate a research-like experience into an introductory course in data analysis or statistics. The labs leverage their natural curiosity and desire to explain the world around them so they can experience both the power and limitations of statistical analysis. They are an excellent way for instructors to transition from textbook problems that focus on procedures to a deeper learning experience that emphasizes the importance of proper experimental design and understanding assumptions. While playing the role of a researcher, students are forced to make decisions about outliers and possibly erroneous data. They experience messy data that make model assumptions highly questionable. These labs give students the context for understanding and discussing issues surrounding data cleaning and model assumptions, topics that are generally overlooked in introductory statistics courses.

## Conflict of Interest Statement

The authors declare that the research was conducted in the absence of any commercial or financial relationships that could be construed as a potential conflict of interest.

## Footnotes

^1^In introductory courses, graphical methods and rules of thumb are usually used to detect outliers. Formal tests such as the Ueda method and Carling’s method could be used in more advanced courses.

^2^While the log transformation is helpful, both groups still show some evidence of lack of normality. Other, less common, transformations did better fit the normality assumption, however, the resulting *p*-values were fairly similar.
